# At-home feasibility trial of genital nerve stimulation to modulate neurogenic bowel dysfunction in individuals living with spinal cord injury: a protocol

**DOI:** 10.3389/fneur.2026.1922192

**Published:** 2026-08-21

**Authors:** Robert Hoey, Amy Binko, Dennis Bourbeau, Ronnie Fass, Doug Gunzler, Laney Korty, Anvi Kshirsagar, Mayson Moore, Megan Moynahan, Megan Hammond Nechols, Benjamin Petrie, Ed Stitts, James Wilson, Kimberly D Anderson

**Affiliations:** 1 MetroHealth System | Case Western Reserve University School of Medicine, Department of Physical Medicine and Rehabilitation, Cleveland, OH, United States; 2 Veteran Affairs Northeast Ohio Health System, Cleveland, OH, United States; 3 MetroHealth System | Case Western Reserve University School of Medicine, Department of Gastroenterology and Hepatology, Cleveland, OH, United States; 4 MetroHealth System | Case Western Reserve University School of Medicine, Department of Medicine, Cleveland, OH, United States; 5 Department of Population and Quantitative Health Sciences, Case Western Reserve University School of Medicine, Cleveland, OH, United States; 6 Kessler Institute for Rehabilitation, West Orange, NJ, United States; 7 Department of Physical Medicine and Rehabilitation, Rutgers New Jersey Medical School, Newark, NJ, United States

**Keywords:** at-home interventions, electrical stimulation, neurogenic bowel, neuromodulation, spinal cord injury

## Abstract

**Introduction:**

Neurogenic bowel dysfunction is a consequence of almost all spinal injuries. Primary management is via conservative treatment methods with few intermediary interventions available prior to surgical management. This protocol describes a trial to determine if genital nerve stimulation (GNS) can reliably be delivered at-home for the purpose of modulating neurogenic bowel dysfunction in people living with spinal cord injury (SCI).

**Methods and analysis:**

A total of 12 participants, aged 18 years or older with any severity of a traumatic SCI (at least 6 months post-injury) at or above the neurological level of T12 with severe neurogenic bowel dysfunction will be enrolled, randomized, and perform GNS or sham stimulation 6–8 h per day for 4 weeks. Daily bowel and stimulation diaries will be filled out by participants or caregivers to provide qualitative and feasibility data. Anorectal manometry (ARM) assessments will be performed at Baseline (before stimulation), at the End of Treatment (the next day after cessation of at-home stimulation), and at the End of Study (after 2 weeks of no stimulation). ARM assessments provide quantitative data for changes in anal sphincter and rectal function due to stimulation. Self-report questionnaires will be completed at each ARM assessment regarding neurogenic bowel dysfunction and impacts of bowel function on quality of life.

**Discussion:**

Outputs from this trial will inform research and clinical practice on the preliminary neuromodulatory effects of GNS on SCI-induced neurogenic bowel dysfunction and will guide the development of a properly powered efficacy trial testing GNS as an intermediary intervention.

**Ethics and dissemination:**

The study was approved by the MetroHealth Institutional Review Board (STUDY00000717) and registered at clinicaltrials.gov prior to enrollment of participants. Dissemination of results will be through presentations at academic meetings and in the community, and peer-reviewed medical journals.

**Clinical trial registration:**

https://clinicaltrials.gov/study/NCT06836739, identifier NCT06836739.

## 1 Introduction

Neurogenic bowel dysfunction (NBD) is a highly prevalent and clinically significant consequence of spinal cord injury (SCI), affecting the majority of individuals living with SCI regardless of injury level or severity. Epidemiological estimates suggest that approximately 261,168-399,079 individuals in the United States live with traumatic SCI, with roughly 18,482 new cases annually, and bowel dysfunction represents one of the most impactful long-term complications in this population (1, 2).

NBD arises from disruption of the complex neural control of colorectal function, which involves coordinated interactions between the enteric nervous system, spinal reflex pathways, and supraspinal control centers (3). Damage to the spinal cord impairs voluntary control, alters reflex activity, and disrupts colonic motility, resulting in a spectrum of symptoms including constipation, fecal incontinence, prolonged bowel care routines, and impaired evacuation (3, 4). These symptoms substantially reduce quality of life and are consistently ranked by individuals with SCI as among the most burdensome complications, often exceeding mobility impairments in perceived impact (5, 6).

The clinical presentation of NBD depends largely on the neurological level and completeness of injury. Injuries above the sacral spinal cord (typically above S2–S4) result in an upper motor neuron bowel syndrome, characterized by preserved spinal reflexes but loss of supraspinal modulation (3, 7). This leads to increased colonic and anal sphincter tone, reduced coordinated peristalsis, and impaired relaxation during defecation. Clinically, this manifests as constipation, stool retention, and reflex-mediated defecation with potential episodes of incontinence (3, 7). In contrast, injuries involving the sacral cord or cauda equina produce a lower motor neuron bowel syndrome, in which reflex activity is diminished or absent (3, 7). Loss of external anal sphincter tone and impaired reflex defecation contribute to fecal incontinence and incomplete evacuation. Compared to upper motor neuron injuries, lower motor neuron dysfunction is often associated with slower colonic transit and greater difficulty maintaining continence (3, 8). Beyond segmental reflex changes, SCI also induces widespread alterations in gastrointestinal physiology, including delayed colonic transit, reduced rectal compliance, and impaired sensory perception (9). These changes reflect both intrinsic enteric alterations and disrupted autonomic regulation, particularly involving sympathetic and parasympathetic pathways (3, 9).

The consequences of NBD extend beyond gastrointestinal symptoms. Individuals with SCI often require prolonged bowel care routines, frequently exceeding 30–60 min per session, and rely on interventions such as suppositories, digital stimulation, or manual evacuation (6, 10). These management strategies can be physically and psychologically burdensome, contributing to reduced independence and social participation (6). Importantly, bowel dysfunction is a leading cause of medical complications after SCI, including fecal impaction, megacolon, hemorrhoids, and autonomic dysreflexia (3, 10). It is also a major contributor to hospital readmissions in this population (11). The chronic nature of these complications underscores the need for improved therapeutic strategies.

Management of NBD is primarily conservative and focuses on establishing predictable bowel routines. Standard interventions include dietary modification, oral laxatives, rectal stimulants (e.g., suppositories), digital rectal stimulation, and manual evacuation (3, 10–13). In refractory cases, more invasive approaches such as transanal irrigation, sacral nerve stimulation, or surgical interventions (e.g., colostomy) may be considered (14). Sacral nerve stimulation (15) and posterior tibial nerve stimulation (16) have been shown to improve neurogenic bowel symptoms but involve surgical implantation of a device or invasive electrode placement, respectively. While these strategies can provide symptom relief, they are often insufficient to fully restore bowel function or quality of life. Surveys consistently report dissatisfaction with current treatments, highlighting the need for novel therapeutic approaches (5, 6).

Given the shared neural circuitry between bladder and bowel control, neuromodulation strategies developed for neurogenic bladder dysfunction have been explored for NBD. Peripheral nerve stimulation, including stimulation of pudendal or genital afferents, has demonstrated the ability to modulate spinal reflex pathways and influence pelvic organ function (17, 18). In particular, genital nerve stimulation (GNS), which targets sensory branches of the pudendal nerve, has been shown to suppress detrusor overactivity and reduce urinary incontinence in individuals with SCI (17, 18). Because bladder and bowel reflexes share overlapping spinal circuits, it has been hypothesized that similar stimulation paradigms may influence bowel function (19).

However, direct evidence for the efficacy of GNS in treating NBD remains limited, and current data should be considered preliminary. Small pilot studies using acute stimulation procedures suggest potential benefits in SCI (20, 21). Chronic GNS has been tested in several at-home pilot studies targeting fecal incontinence in people without SCI. At-home, chronic, GNS has shown promise in symptom improvement with reduced number of incontinence events in some participants (22, 23), and a ≥50% reduction in symptoms (24). These studies suggest that using at-home GNS procedures over a chronic period may improve fecal incontinence and is consistent with a recent review and meta-analysis of GNS and bowel function (25). Therefore, studies using at-home chronic GNS trials in people living with SCI may provide improvement in bowel symptoms in this population. Limitations regarding the underlying mechanism for fecal incontinence (idiopathic vs. neurogenic) necessitates direct investigation of potential benefits in the SCI population.

Because the needs of a non-spinal cord injured individual can differ markedly from those of a person living with SCI, it is necessary to conduct a feasibility trial to understand and limit participant burden by using decentralized strategies, including at-home stimulation. This strategy will inform investigations as to the best methods and common pitfalls to avoid before investing significant time, effort, and finances into pivotal efficacy trials that are impractical to perform with the SCI population. Therefore, this feasibility trial was designed to elucidate the specific needs of people living with SCI related to the administration of at-home GNS prior to efficacy investigations.

The primary objective of the proposed study is to determine the feasibility of daily application of GNS at-home in people living with SCI. Key feasibility measures relate to recruitment of appropriate participants, dosage (target vs. sham), and adherence. A secondary objective is to evaluate effect sizes in anorectal physiology parameters as well as symptom-based participant-reported outcomes.

## 2 Methods and analysis

### 2.1 Trial design and setting

**Figure 1:**
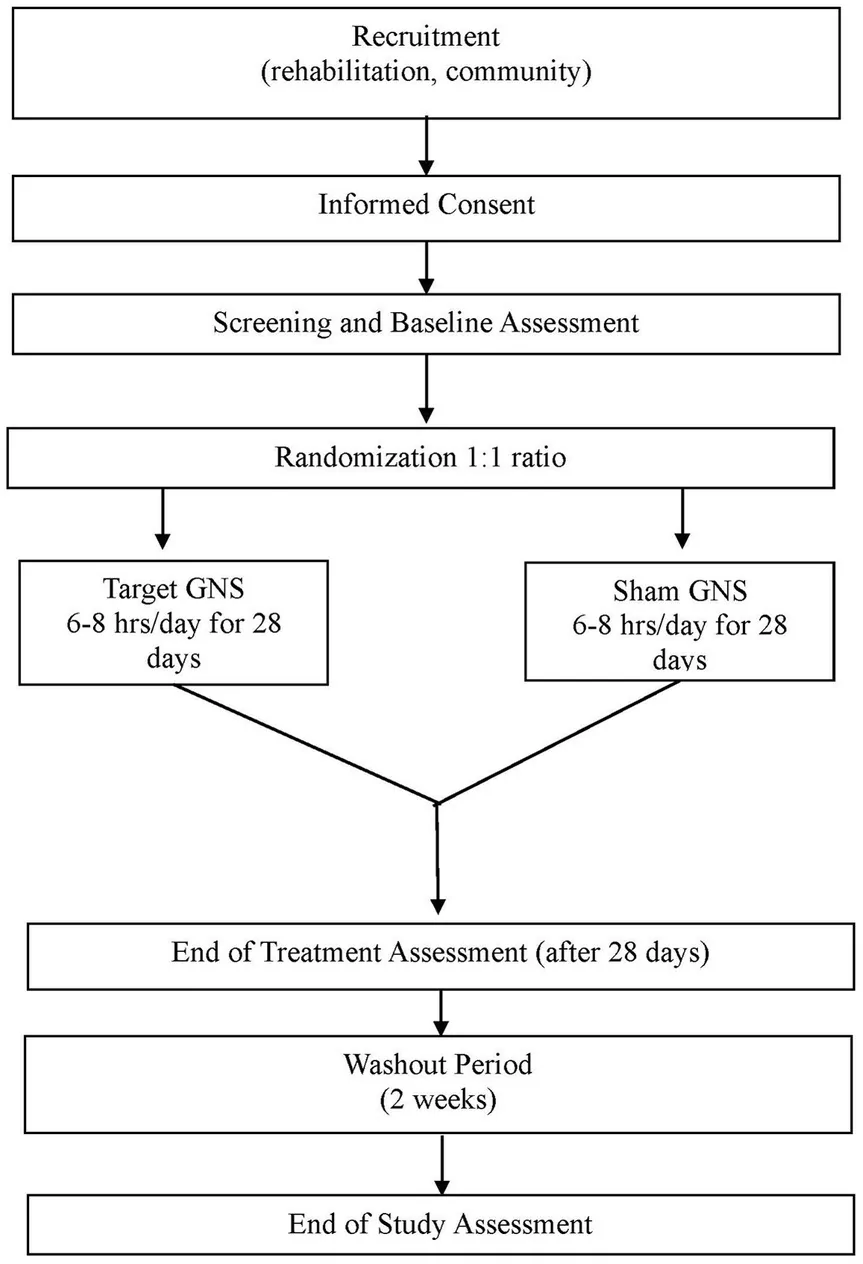
Study flow chart. GNS, genital nerve stimulation.

This is a single site, feasibility trial, conducted in an academic hospital setting. A randomized, parallel group study design will be used for the at-home daily use of GNS, which emulates a mock efficacy trial design. Participant blinding will be utilized to ensure participant reported outcome measures are not impacted. The mock efficacy trial design includes Baseline assessment visit, randomization to GNS or sham, treatment of 6–8 h daily for 4 weeks, End of Treatment assessment visit, washout of no stimulation for 2 weeks, and End of Study assessment visit (Figure 1). Enrollment was opened on 1 March 2025, recruitment is ongoing, and three participants have been enrolled.

### 2.2 Participants and sample size

#### 2.2.1 Eligibility criteria

Eligible participants will be over 18 years of age, able to provide informed consent, have a traumatic SCI at neurological level T12 or above, and be American Spinal Injury Association Impairment Scale (AIS) grade A–D. Participants will also be at least 6 months past the date of injury and score in the severe category (≥14) on Neurogenic Bowel Dysfunction Score (26). Finally, possible participants must respond to GNS, which will be determined by 20 Hz stimulation causing reflex contraction of the anus (pudendo-anal reflex, aka anal wink reflex). The threshold of this reflex activation will be used to determine stimulation parameters during the intervention. Exclusion criteria are enrollment in other functional electrical stimulation trials, females who are pregnant or trying to become pregnant during the study, presence of other implanted devices that may interact with GNS (i.e., pacemaker, defibrillator, baclofen pump), and the presence of health conditions that may make participation unsafe as determined by the study doctor. Pregnancy is confirmed verbally during screening and any participant who becomes pregnant during the study will be withdrawn, as per Institutional Review Board requirements. Concurrent neurogenic bladder dysfunction is neither an inclusion nor exclusion criterion for this study. It is common for there to be both bladder and bowel dysfunction after SCI, therefore a column was included in the bowel diary to capture changes in bladder function rather than make it a criterion for participation.

#### 2.2.2 Sample size

The purpose of a feasibility study is to ask and answer questions that will aid in the planning of a large-scale study, and as such, are not intended to determine statistical significance. Therefore, traditional sample size calculations to power efficacy are inappropriate (27, 28). Rather, sample size should be large enough to yield useful data to answer the feasibility questions. Critical feasibility questions for a future efficacy trial of daily at-home GNS revolve around whether the stimulation protocol can be delivered at the prescribed dose, including sham stimulation, can participants remain blinded to stimulation group, and will participants complete the study. Based on those feasibility questions, we will target enrolling 12 responders to GNS over a 2 year period. The number of participants in this pilot trial is based on previous literature (29, 30) showing a sample size of 12 per group is sufficient for pilot investigations. Because the primary outcomes are feasibility measures that do not depend on stimulation frequency, all 12 participants will be compared as a single group. All other outcomes are considered exploratory in nature and therefore specific sample sizes are not powered to determine efficacy or effect size for measures after stimulation group assignment (i.e., *n* = 6 per group).

#### 2.2.3 Recruitment and retention

A convenience sample of participants will be purposively recruited based on the eligibility criteria. The study site is within a clinical system that provides a continuum of care for SCI and regularly interacts with individuals living with SCI. The study site also has an existing research infrastructure. Recruitment will utilize multiple strategies and be from multiple locations, including the internal clinical system, the local Veterans Affairs healthcare system, outreach to collaborative research centers across the country, and outreach to multiple SCI community-led organizations. Common barriers to participation in research studies are transportation and accessible lodging. To eliminate these barriers, funds are budgeted for transportation, travel, and accessible housing on the research campus. Compensation for participation is provided at the end of each assessment visit based on a rate of $20 per visit hour. Retention strategies will include weekly communication facilitated by the study coordinator to build rapport and offer support, giving participants a stimulation unit, leads, and electrodes to keep at the end of study participation, and giving each participant a summary report of his/her individual data results a few weeks after the end of study participation.

### 2.3 Randomization and intervention

Randomization to group assignment will not be made until all Baseline assessments are completed. If a participant is not eligible for the study (screen failure) all study procedures are stopped and their data will not be used in subsequent analysis; this includes non-responders to GNS. Participants will be randomized in a 1:1 ratio to either target stimulation (*n* = 6) or sham stimulation (*n* = 6) based on a list created by the study statistician prior to enrollment of the first participant. Randomization was done using a random number generator attached to a list of all 12 possible assignments to determine the order and kept on a master enrollment list. Therefore, group assignments were randomly assigned based on successful screening into the study without stratification or blocking. Allocation will remain concealed from study staff performing recruitment and enrollment. Recruitment and enrollment are performed by the clinical research coordinator. Group assignment allocation is not performed by the clinical research coordinator. Rather, it is performed by the study investigator. For both groups, all participants will maintain their usual bowel routine and nutritional habits. Participants in both groups will perform stimulation daily for 6–8 h, between bowel-emptying episodes, and at the same time each day. Stimulation will be applied for 28 consecutive days.

Genital nerve stimulation will be delivered using the Ultima Neo TENS unit (Pain Management Technologies, Akron, OH). This is an FDA-approved Class II medical device. Stimulation will be administered via non-invasive surface skin electrodes. In men, two surface electrodes (1 cm diameter) will be placed on the dorsum of the penile shaft 2 cm apart. In women, one surface electrode will be placed near the clitoris and a second surface electrode will be placed on the labia majora or inner thigh. The electrodes will be connected to an electrical stimulator device.

Stimulation waveforms consist of biphasic, charge-balanced square pulses with a pulse width of 0.2 ms and delivered at 20 Hz. Stimuli will be applied over a range of amplitudes to determine the threshold of stimulation for producing reflex contraction of the anal sphincter (the pudendo-anal reflex). Stimulation will be ramped up from threshold level at week 1, to 1.5 × threshold at week 2, to 2 × threshold (target) at weeks 3–4. The typical stimulation range is 20–40 mA and has been shown to be well tolerated by individuals with sensation (31).

Sham stimulation will be applied in the same manner as target stimulation, except that the stimulation frequency will be 1 Hz and the stimulation amplitude will be set to a limit that is at the threshold of perception if individuals have sensation, or 10 mA if they do not have sensation. Stimulation of the genital nerves at 1 Hz and low amplitude is not expected to have an effect on pelvic autonomic functions as it did not change bladder function (31).

During the screening procedure the participants will be exposed to both active and sham stimulation frequencies, but not told which is active vs. sham. Additionally, during the Baseline ARM test the participant will only be exposed to the stimulation frequency he/she will be self-delivering at home. In this way, even if participants can detect the difference between frequencies, they will not know what that difference means until after the washout ARM session, thus maintain blinding. Participants will only be told which group they were a part of during the End of Study debriefing.

All participants will be instructed on how to put on and use the Ultima Neo TENS device, will be provided with a visual instruction guide to take home, and will be given electrodes and a battery charger. The research team will set the parameters of the Ultima Neo TENS device to deliver target or sham stimulation while keeping the participant blind to the settings by masking the stimulation parameter display settings (frequency and pulse width) with electrical tape. This process leads to participant, but not investigator, blinding throughout the study. The data analyst will be blind to participant group assignment throughout the study and therefore leads to a double-blinding of participant and study data analyst. ARM data will not be interpreted in real time during the procedure. Rather, data will be extracted from ARM traces using automated custom software to avoid confounding of manual data extraction from an unblinded assessor.

Any participant who answers ‘No’ or ‘I do not know’ to the study exit question ‘Do you think you know what study group you were randomized to?’ will be classified as blinded. Any participant who answers ‘Yes’ to this question will be classified as unblinded. We will use the James Blinding Index (32) to evaluate trial masking along with the 95% confidence interval. A value greater than 0.50 will be considered indicative of acceptable blinding. To account for the potential of sacral sensory status leading to unblinding, we will perform a subgroup analysis of the blinding index based on sensory status. Participants with no sensation in the S4–5 region are classified as AIS grade A; participants with any degree of sensation in S4–5 are classified as AIS B, C, or D (dependent upon their motor status). For the subgroup analysis, participants that are AIS A will be put in the ‘No sensation’ subgroup and participants that are AIS B–D will be put in the ‘Sensation’ subgroup. The James Blinding Index and 95% confidence interval will be computed for each subgroup. Comparison between the two subgroups will be performed qualitatively by assessing for overlap in confidence intervals. If the index for the Sensation subgroup drops below 0.50 or clearly diverges from the no sensation subgroup, it will be an indicator that preserved sensation in S4–5 is a mechanism for unblinding.

### 2.4 Assessments

#### 2.4.1 Primary feasibility measures

The primary endpoint of this trial is feasibility. Table 1 lists the feasibility questions, measures, and benchmarks for success. These questions focus on the delivery of the at-home stimulation (9 of 12 enrolled each receive ≥142.8 h of stimulation), ability to keep participants blinded (James Blinding Index >0.50), and retention (9 of 12 participants stay enrolled until the end of the study and complete all assessments). Additionally, it is important to be able to collect participant feedback on the ease or difficulty of using the Ultima NEO TENS unit and applying genital nerve stimulation at home. This is important feasibility information that will be collected using an open-ended questionnaire with prompts probing for any difficulty placing electrodes, any difficulty keeping electrodes in place, any need for further training on the device once home, any device malfunctions, any need for replacement components or electrodes. A study exit questionnaire will be used to determine whether participants remain blinded throughout the study and any perspectives on their experience while in the study. This questionnaire asks whether the participant completed the study, the reason for not completing the study if appropriate, whether the participant thinks they know which group they were randomized to, if the answer is ‘yes’ the participant states which group, and why they think they know to which group they were randomized.

**Table 1:** Feasibility questions, measures, and benchmarks of success.

Feasibility question	Feasibility measure	Quantitative benchmark of success
Can the stimulation protocol be delivered?	Proportion of enrolled who complete the stimulation protocol (treatment arm; sham arm); number of deviations/arm; reasons for deviations	9 of 12 enrolled each receive ≥142.8 h of stimulation
Can participants remain blinded?	Number of occurrences of unblinding; reasons for unblinding	James Blinding Index >0.50
Will participants complete the study?	Retention rate; reasons for dropout; proportion of planned assessments completed	9 of 12 participants stay enrolled until the end of the study and complete all assessments

#### 2.4.2 Secondary efficacy measures

The goal of genital nerve stimulation is to modulate the defecation reflex. Therefore, the preliminary measure of efficacy will be anorectal reflex activity as measured by anorectal manometry (ARM). ARM is the gold standard clinical tool for investigating anorectal function (33). It is widely available and very well established. Recent efforts have been made to standardize ARM testing protocols so that results can be categorized (34). The London Classification (34) protocol timeline will be used in ARM testing in this experiment and can provide the following data, most of which will be collected in this trial:

1. Anal sphincter function (assessed by measuring resting sphincter pressure and squeeze sphincter pressure).
2. Rectoanal reflex activity (a measure of fecal incontinence when (1) the anal sphincter pressure is less than the intra-abdominal pressure due to rectoanal inhibitory reflex reducing anal sphincter pressure from rectal stimulation, i.e., presence of stool; or (2) the sphincter does not contract during rapid increased abdominal pressure, i.e., during a cough).
3. Rectal sensation (assessed by measuring the smallest volume of fluid that elicits a first sensation, a desire to defecate, and the maximal tolerable volume of fluid).
4. Changes in anal and rectal pressures during attempted defecation (a measure of dyssynergia and is quantified via the defecation index, which equals the maximum rectal pressure during attempted defecation/minimum anal residual pressure during attempted defecation). This measure was omitted because catheter movement can alter rectal activity due to mechanical stimulation of the rectum, thus producing an artifact. Because multiple runs of the timeline will be done in one session it was decided that the catheter will be left in place, to reduce mechanically stimulated rectal activity, and the defecation attempt will not be performed. Additionally, subjecting participants to testing in which an artifact would be produced would increase the time burden and, in our opinion, be unethical.
5. Rectal compliance/rectal pressure (a measure of capacity and distensibility).

This version of ARM collects many of the same data as the standard clinical ARM assessment elucidated in the London Classification system. Notably, the timeline of actions is consistent and so many of the results will be applicable. Although, it is worthwhile to note the differences and understand how this may influence interpretation of the results. First, the collection of continuous rectal pressure rather than a pull-through maneuver was chosen to reduce mechanically stimulated rectal activity especially across multiple runs of the timeline in one session. Additionally, the concurrent collection of rectal and sphincter data provides time-locked activity in both systems and adds dynamic data to aid interpretation of function. Second, omission of the defecation component may limit the interpretation of the functional aspect of difficulty with defecation in this population. It is clear from previous work that difficulty with defecation is common after SCI and it is not clear if the increased variability and time burden of this test (explained above) would improve interpretation of the functional outcomes. Third, the averaging of 3 circumferential sensors for anal sphincter pressure is less than the 4 typically used. This was due to the limit of our system when the fluidic pressure channel was added. This change limits the interpretation of anal quadrant differences. Although, because of the lateral recumbency positioning there is increased pressure in the inferior sensor due to gravity on the catheter. Therefore, while some data will be lost, there may be limited utility in a quadrant analysis due to mechanical forces on some sensors. Fourth, the combination of air-charged sphincter pressure sensors with a fluid rectal channel differs from the London standard but was done using an off-the-shelf Laborie clinical unit and is consistent with accurate pressure recording. Additionally, fluidic pressure measurement is more accurate than air-charged as fluid is non-compressible. Therefore, pressure changes at the balloon will be transmitted to the sensor with greater fidelity than air-charged systems due to compression of air within the balloon. When taken together, these changes amount to a modified London Classification assessment that is appropriate for measuring these measures in the SCI population, with our multiple run protocol. The omission of some facets (defecation test) and the modification of rectal measurement (continuous vs. pull-through, fluidic pressure measurement) may make the end results less uniform with the diagnostic component of the London Classification system in some regards. Although, we feel the addition of more accurate fluidic rectal measurement, dynamic and time-locked responses of the anal sphincter and rectum, and inclusion of validated questionnaires on bowel function and quality of life make this protocol appropriate for this population.

In addition to using ARM to understand how GNS modulates bowel neurophysiology, it is also important to evaluate how it may impact clinical characteristics, function, and quality of life. A clinical exam will be conducted by a clinician and include an abdominal examination, evaluation of sacral reflexes, anal sphincter tone, sensation, and voluntary contraction. So as not to duplicate assessments, anal sphincter tone, sensation, and voluntary contraction will be captured during the International Standards of Neurological Classification of SCI (ISNCSCI) at screening (35). The clinical exam will be performed by an SCI-specialized clinician. Data captured during the clinical exam will provide a clinical characterization of bowel dysfunction.

The International SCI (ISCI) Bowel Function (BF) Basic Dataset (BDS) version 2.1 (36) incorporates the Neurogenic Bowel Dysfunction Score (NBDS) as well as information about gastrointestinal or anal sphincter dysfunction unrelated to SCI, surgical procedures on the gastrointestinal tract, defecation methods, and bowel care procedures, the need to wear external continence products, and presence of abdominal pain or discomfort. The NBDS was developed specifically for SCI and measures the degree of neurogenic bowel dysfunction symptomology and the impact on quality of life (QoL; 26). There are 10 items and they are symptom-based and weighted by the impact on QoL. The total score can be interpreted in the following categories:

0–6 very minor neurogenic bowel dysfunction.
7–9 minor neurogenic bowel dysfunction.
10–13 moderate neurogenic bowel dysfunction.
14–45 severe neurogenic bowel dysfunction.

The NBDS has undergone multiple validation studies (37, 38).

The SCI-QoL Bowel Management Difficulties measure documents difficulties people living with SCI have with bowel management, including feelings of distress associated with bowel problems they experience in daily life (39). The items focus on issues related to fecal incontinence and the psychosocial consequences of fecal incontinence, which is different than the ISCI BF BDS v2.1 and why it was selected as an additional secondary outcome measure. Validity and reliability have been demonstrated (39). The full scale is composed of 26 items, but a computer assisted technology version and 9-item short form version have also been created. The short form version will be used in this trial and is available for download from Assessment Center. Each item has a response option based on a 5-point Likert scale ranging from 1 ‘never/not at all’ to 5 ‘always/very much’. Higher scores represent greater difficulty managing bowel problems.

A bowel diary is a means for participants to record daily bowel activities. Parameters to be recorded include stool type [using the Bristol Stool Scale; (40)], amount, planned emptying episodes (including time), incontinence episodes, sense of urgency, straining, all medications, and complications such as nausea or hemorrhoids (41). These data will characterize each participant’s bowel management routine, daily habits, and gastrointestinal symptoms. Additionally, to capture any changes in bladder or sexual function, participants report daily on any changes in those functions within the bowel diary.

Participants will also complete a stimulation diary during the at-home stimulation period. The components include when stimulation was performed, the stimulation parameters, and notes to include any issues or perceptions of the stimulation. This allows for the determination of the amount of time the participant was exposed to stimulation and the parameters of that stimulation (dose) as well as any problems they encountered.

Additional data collected will include demographics (gender, age, race, ethnicity, education, primary occupation, marriage status, household size, and area of residence); cause of spinal injury; and brief medical history; and concomitant medications.

### 2.5 Adverse event monitoring

All data will be monitored for safety. This includes from the time informed consent is provided through the End of study visit. Safety data will be collected at the three study visits as well as via weekly telephone calls with participants. Adverse events will be documented on an adverse event log and evaluated for severity, expectedness, and relatedness following FDA and IRB guidelines.

Possible adverse events include tissue irritation, tissue burn, uncomfortable physical sensations, uncomfortable psychological events, and autonomic dysreflexia (AD). AD is defined as a rise of 20 mmHg systolic pressure above baseline. GNS alone does not typically cause AD and can reduce AD triggers by reducing spastic contractions in the bladder (31). A common AD trigger is rectal distension and the most likely period in the study to induce an AD episode is during the rectal sensation component of the ARM test when the balloon is being inflated. Blood pressure will be measured throughout the ARM procedure (every 5 min), with particular attention paid to readings during balloon inflation. If there is a rise in systolic pressure of 20 mmHg, the procedure will be paused, and blood pressure will be monitored to see if the rise in systolic blood pressure resolves. The study nurse will be responsible for monitoring and managing AD symptoms during the ARM under the oversight of the study physician.

A Medical Monitor will monitor for clinical safety. Adverse events will be reviewed by the Medical Monitor on a quarterly basis with the study staff (additional *ad hoc* review can occur if necessary for any individual adverse event). The Medical Monitor will be a physician with board certification for SCI medicine and have expertise in SCI-induced altered sensations, skin safety, and AD. The Medical Monitor will examine all adverse events for reportability (unexpected, related or possibly related, AND resulting in increased risk). Non-reportable adverse events will be documented internally and included in continuing institutional reviews.

### 2.6 Clinical equipment

A height-adjustable mat table that is large enough to provide room for the participant to move from side to side as necessary for procedures or comfort will be used for examinations. A 4″ gelfoam mattress pad will be placed on the mat table for comfort during the ARM test.

The clinical urodynamics testing unit (Laborie, Portsmouth, NH) with an anorectal manometry configuration will be used for ARM testing. It uses an air-charged pressure system (TDOC) as well as a fluidic pressure system. The ARM catheter (Laborie, CAT003) has circumferential (4 sites) pressure sensation to detect anal canal function, three of which will be used and averaged to account for the effect of catheter placement and gravity on pressure values. The fluidic system is attached to the rectal balloon to simultaneously record both rectal and sphincter pressures during the ARM test. Fluidic rectal pressures are used for collecting rectal pressures simultaneously with anal sphincter pressures. The rectal balloon catheter is connected to the Laborie unit via urodynamics measurement tubing (Laborie, TUB101) and a transducer (Laborie, DIS130). The typical method for obtaining rectal pressures is to perform a “pull through” procedure. Manual stimulation to the rectum via the “pull through” procedure could influence the results by the rectum having a response to that manual stimulation. Therefore, to capture the dynamic coordination of both systems we will continuously monitor rectal pressures during testing as an additional pressure data channel.

Natus electrodes (Natus, 019-400400) will be used to apply stimulation. These electrodes will be used due to their smaller size which is more appropriate to deliver stimulation to the genital nerve receptive field. To connect these electrodes to the TENS unit, we modified the electrode cable with connectors that are compatible with Natus electrode connection (Neurospec, NS7486).

### 2.7 Lived experience consultants

Our study team has two people that are living with SCI as consultants. These individuals participate in team meetings and provide an important perspective on all aspects of the study to ensure that our methods and procedures are reasonable and applicable to those living with SCI. This input ranges from contributing to study design and evaluating study documents to testing procedures to ensure that participants who enroll in the study will be able to complete all procedures. These relationships have been invaluable in translating knowledge from idea generation to practical applications in the lives of people living with SCI.

### 2.8 Data management and analysis

Participants will be assigned a study code upon study entry. The linkage between the study code and participant identity will be stored in a secured REDCap database. Paper consent forms will be stored in a locked cabinet in a locked room with only study staff access. The study code will be used on all case report forms. Data will be evaluated for quality assurance in real time by the Principal Investigator. After which all data will be entered into a separate secured REDCap database for storage by the study coordinator. Quality assurance procedures will be repeated for all data entered into the database.

Primary data analysis will be conducted based on the feasibility benchmarks listed in Table 1. If all the quantitative benchmarks of success are met, it will be considered feasible to pursue an efficacy trial in the future based on the current design. If the quantitative benchmarks are not met, that will be indicative of design parameters that need to be modified prior to launching an efficacy trial. Because participant withdrawal is a feasibility measure, any participant that withdraws will be included in the feasibility analysis. Only existing data will be used for exploratory analysis and missing data will be handled as described below.

During ARM testing we will document responses of tissues when participants are trying to perform specific actions (i.e., squeeze, push, etc.). This data will allow for conclusions to be drawn about the effect of an extended administration of GNS (4 weeks, 6–8 h per day) on defecation, continence, anal sphincter tone, rectal pressures, reflex activity, and sensation thresholds. Furthermore, we can determine if the sham stimulation chosen for this feasibility trial is having an effect on function. We will calculate change scores (i.e., difference scores) across each successive time point (Baseline to End of Treatment to End of Study) for all ARM data parameters. We will calculate descriptive statistics (e.g., mean and 95% confidence interval, median, and interquartile range) to summarize these change scores across time. Graphical summaries, including box-and-whisker plots and histograms, will be used to visualize the distribution of ARM parameters and change scores, assess potential outliers, and evaluate individual patterns of change over time. These visualizations will complement descriptive statistics and aid in the interpretation of treatment and sham group responses.

Additionally, we will report the population demographics, adverse events, medications, and all outcome measures. To do so we will calculate descriptive statistics for each outcome (e.g., frequency counts, percentages, means, medians, standard deviations, interquartile ranges, confidence intervals). We will report overall results within each group (sham, GNS) as well as differences across groups. We will not test for statistical significance of efficacy because that is not the intent of this study. In addition to using the descriptive statistics, we will perform a sensitivity analysis to inform a future efficacy trial. The literature suggests using the feasibility study’s upper limit of the 80% confidence interval for the standard deviation when calculating the sample size needed for the future efficacy trial (28). We will analyze the primary ARM results with confidence intervals at 75, 80, 85, 90, and 95% to get a preliminary, cautious view of efficacy/effect (28). This thorough sensitivity analysis will inform a sample size calculation for a future trial.

Because this is a feasibility study with a planned sample size of 12 participants (6 per group), missing data will be summarized descriptively. Missing data are expected to be minimal in this study. We will report the number and percentage of missing observations for each outcome and document the reasons for missing data when available (e.g., participant withdrawal, missed visit, equipment failure). Analyses will be conducted using available-case data, with no imputation of missing values. Change scores will be calculated only for participants with measurements at the relevant time points. For each outcome and assessment time point, we will report the number of participants contributing data overall and by treatment group to provide transparency regarding the completeness of the data. Rates of missing data, participant retention, and adherence to the intervention and assessment schedule will be considered key feasibility outcomes and will be used to inform study procedures, estimate attrition for future studies, and guide the design of a subsequent adequately powered efficacy trial.

## 3 Discussion

This protocol design is a comprehensive and participant-centered framework for evaluating NBD following SCI, integrating objective physiological measures, participant-reported outcomes, and feasibility metrics. The primary strength of this design lies in the deliberate collection of multidimensional data to address the complexity and heterogeneity of bowel dysfunction in this population.

A central methodological consideration is the integration of ARM testing with validated bowel function and quality-of-life questionnaires. While ARM provides detailed, real-time physiological measurements, its clinical interpretation in SCI remains uncertain, particularly when compared with able-bodied reference values. ARM results provide an objective measure of what the participant’s body is doing at that moment. This data is valuable on its own, and some conclusions can be drawn based on these measures (i.e., resting pressures, changes during actions). As yet, it is unclear whether it is appropriate to compare the ARM results of those with SCI to an able-bodied standard to determine dysfunction in a person with SCI. It could be that after an injury there is a different physiological standard that would produce appropriate function. For example, because the anus does not have to maintain continence against gravity, in those who use wheelchairs, the resting anal pressure required to maintain continence may be lower. The issue of what standard should be used in SCI research also applies to the effect of a targeted intervention. This protocol addresses this clinical interpretation limitation by explicitly linking objective measures to participant-reported outcomes, allowing for identification of physiologically meaningful targets that correspond to functional improvements rather than normative comparisons. This approach is expected to improve interpretability of ARM data and enhance its translational relevance.

It is unknown whether sham stimulation is inert. It may provide afferent stimulation to the spinal cord that could drive plasticity during the four week stimulation period. When coupled with repeated ARM test sessions (which include rectal distension and related afferent signals from performing actions) there may be positive effects on function that cannot be predicted *a priori*.

The study also emphasizes the importance of capturing a broad range of contextual variables, including injury characteristics, medication use, and lived experience. These data are expected to support exploratory analyses aimed at identifying patterns of dysfunction and potential predictors of responsiveness to intervention. Collecting Baseline data prior to stimulation will inform about the specific dysfunction associated with clinical characteristics and other associated data being collected. For example, do people with similar levels of injury have similar dysfunctions? The capability to predict whether a person would have a favorable response to certain treatments would lead to better proactive interventions regarding bowel dysfunction after SCI. Given the known heterogeneity in SCI presentations, this breadth of data collection is necessary to inform future stratification strategies and to support the development of individualized therapeutic approaches.

Feasibility and accessibility considerations are embedded throughout the study design. Barriers to participation, such as transportation and accessible lodging, are proactively addressed to enhance recruitment and retention. In addition, the inclusion of lived-experience consultants in study development ensures that procedures are acceptable and relevant to the target population. These elements are critical for ensuring that the protocol can be implemented in a manner that is both ethical and scalable in future trials. By incorporating participant responses into the data analysis, we can determine which changes in ARM results are improving the functional aspects investigated by the questionnaires (i.e., toileting time, incontinence, quality of life), rather than comparison to a standard. Therefore, we can utilize the participant’s expertise to understand appropriate targets for the objective measurements. For example, if a person has severe NBD dysfunction and substantially impaired QoL scores, we can determine objective ARM results that cause less NBD dysfunction and improve QoL for that person. These data directly address the feasibility question of compliance and any troubleshooting that may be necessary for this population. Because of the specific needs of those using wheelchairs, it may require a different approach than someone who is ambulatory. Longitudinal data collection, including repeated ARM assessments and at-home stimulation diaries, is expected to provide insight into both short-term and sustained effects of stimulation. These data will also inform feasibility outcomes such as adherence, tolerability, and practical challenges associated with extended stimulation use in individuals with SCI. Such information is essential for designing larger clinical trials and for eventual translation into clinical practice.

From a clinical perspective, this protocol is designed to generate data that may inform more effective management of NBD, a condition that significantly impacts health, independence, and quality of life in individuals with SCI. By identifying relationships between physiological function and participant-reported outcomes, this work aims to support the development of targeted interventions that address both mechanistic and functional aspects of bowel dysfunction. In summary, this protocol provides a structured approach to capturing the complexity of bowel function after SCI. The integration of objective, subjective, and feasibility data is expected to yield insights that extend beyond this study, supporting future research on neuromodulation and contributing to improved therapeutic strategies for NBD.

### 3.1 Limitations

The described protocol requires a participant to have an intact pudendo-anal reflex, intact spinal circuitry below T12, and the ability to respond to GNS. Because of these requirements, the results of this study will not be generalizable to those with lower-motor neuron injuries leading to an areflexic and flaccid anogenital system. This limitation will also influence future studies based on GNS and its effect on bowel function after SCI.

Modification of the London Classification system will limit the comparison between SCI and able-bodied ARM results as they are no longer directly identical. This was done with the needs of the study and population in mind but should be addressed as a limitation with direct comparison of the results.

This study is also further limited by the possibility that sham and repeated manometry may induce plasticity that may not be fully resolved after the two-week washout period. As this is a feasibility trial, we did not design the study to determine the extent of plasticity. A two-week timepoint is sufficient to determine if there is a change from the End of Treatment timepoint, even if plasticity has occurred and has not completely resolved back to baseline. An End of Study ARM result that is less than the Baseline ARM result would suggest plasticity was induced, even if the magnitude and ultimate duration is not fully mapped in this feasibility investigation.

A limitation of the exploratory sensitivity analysis is that we are not pre-specifying one interval, which leaves open the possibility of choosing whichever confidence-interval gives the best results after seeing the data. However, given our study aims, we chose to use the following reasoning instead for our confidence interval reporting. A future efficacy trial is expensive, and because estimates of the standard deviation from a feasibility study are uncertain, presenting several confidence levels illustrates how sensitive the required sample size is to plausible assumptions about variability. The purpose here is transparency and exploratory rather than selecting among those choices.
